# Viability of Quercetin-Induced Dental Pulp Stem Cells in Forming Living Cellular Constructs for Soft Tissue Augmentation

**DOI:** 10.3390/jpm11050430

**Published:** 2021-05-18

**Authors:** Hytham N. Fageeh, Shilpa Bhandi, Mohammed Mashyakhy, Ahmed Al Kahtani, Zahi Badran, Deepak Mehta, Hammam Ibrahim Fageeh, Thodur Madapusi Balaji, Hosam Ali Baeshen, Saranya Varadarajan, A. Thirumal Raj, Vikrant R. Patil, Nishant Vyas, Alessio Zanza, Luca Testarelli, Shankargouda Patil

**Affiliations:** 1 Department of Preventive Dental Sciences, College of Dentistry, Jazan University, Jazan 45142, Saudi Arabia; hfageeh@jazanu.edu.sa (H.N.F.); hafageeh@jazanu.edu.sa (H.I.F.); 2 Department of Restorative Dental Sciences, College of Dentistry, Jazan University, Jazan 45142, Saudi Arabia; shilpa.bhandi@gmail.com (S.B.); dr.mashyakhy@gmail.com (M.M.); 3 Department of Restorative Dental Sciences, College of Dentistry, King Saud University, Riyadh 11362, Saudi Arabia; ahkahtani@ksu.edu.sa; 4 Department of Preventive and Restorative Dentistry, College of Dental Medicine, University of Sharjah, Sharjah 27272, United Arab Emirates; zbadran@sharjah.ac.ae (Z.B.); dmehta@sharjah.ac.ae (D.M.); 5 Department of Dentistry, Bharathirajaa Hospital, and Research Institute, Chennai 600017, India; tmbala81@gmail.com; 6 Department of Orthodontics, College of Dentistry, King Abdulaziz University, Jeddah 21589, Saudi Arabia; drbaeshen@me.com; 7 Department of Oral Pathology and Microbiology, Sri Venkateswara Dental College and Hospital, Chennai 600130, India; vsaranya87@gmail.com (S.V.); thirumalraj666@gmail.com (A.T.R.); 8 Logical Life Science Private Limited, Pune 411041, India; patilvikrant.r@gmail.com (V.R.P.); logicalbiolgy84@gmail.com (N.V.); 9 Department of Oral and Maxillofacial Sciences, Sapienza University, University of Rome, 00185 Rome, Italy; ale.zanza@gmail.com (A.Z.); luca.testarelli@uniroma1.it (L.T.); 10 Department of Maxillofacial Surgery and Diagnostic Sciences, Division of Oral Pathology, College of Dentistry, Jazan University, Jazan 45142, Saudi Arabia

**Keywords:** dental pulp, living cell construct, quercetin, soft tissue, stem cells

## Abstract

Autogenous gingival grafts used for root coverage or gingival augmentation procedures often result in donor site morbidity. Living cellular constructs as an exogenous alternative have been proven to be associated with lower morbidity. With the available background information, the present study aims to assess if quercetin-induced living cell constructs, derived from dental pulp stem cells, have the potential to be applied as a tool for soft tissue augmentation. The characterized dental pulp stem cells (positive for CD73, CD90, and negative for CD34, HLA-DR) were expanded in Dulbecco’s Modified Eagle’s medium (DMEM) supplemented with 10 mM quercetin. The handling properties of the quercetin-induced dental pulp stem cell constructs were assessed by visual, and tactile sensation. A microscopic characterization using hematoxylin and eosin staining, and qRT-PCR-based analysis for stemness-associated genes (OCT4, NANOG, SOX2, and cMyc) was also performed. Dental pulp stem cells without quercetin administration were used as the control. Dental pulp stem cell constructs induced by quercetin easily detached from the surface of the plate, whereas there was no formation in the control cells. It was also simple to transfer the induced cellular construct on the flattened surface. Microscopic characterization of the constructs showed cells embedded in a tissue matrix. Quercetin also increased the expression of stemness-related genes. The use of quercetin-induced DPSC living constructs for soft tissue augmentation could provide an alternative to autogenous soft tissue grafts to lower patient morbidity and improve esthetic outcomes.

## 1. Introduction

One of the main challenges faced in clinical periodontology is restoration of the lost supporting apparatus. Periodontitis, as an inflammatory condition of microbial etiology, disrupts the integrity of the periodontium and causes gingival recession with loss of keratinized tissues. It is also noteworthy that gingival recession could arise from calculus deposition [1], faulty tooth-brushing habits [2,3], and could also be an outcome of anatomic anomalies of the mucogingival apparatus. Whatever the case, elimination of the etiology of gingival recession, coupled with periodontal plastic surgical procedures in indicated cases, is considered the standard management approach for gingival recessions and other mucogingival defects. Root coverage procedures are achieved often by the use of repositioned flap techniques, or by the use of free grafts/biomaterials, such as enamel matrix derivatives, to cover the denuded root and restore aesthetics. In this regard, the most commonly employed technique is to obtain autogenous soft tissue grafts from the patient’s palate. A recently published consensus report, analyzing three significant systematic reviews, concluded that root coverage of single tooth recessions with the use of coronally advanced flaps or connective tissue autografts/enamel matrix derivatives is clinically feasible; however, the coverage of multiple tooth recessions with interdental tissue loss remains a clinical challenge [1]. It is in this scenario that the field of regenerative medicine could come to the rescue. With advancements in the field of tissue engineering and regenerative medicine, cell sheets have been cultured and could be clinically used for the management of gingival recession.

In dentistry, the cultivation of living cells such as fibroblasts and keratinocytes into a scaffolding matrix has gained popularity in the field of periodontal plastic surgery and mucogingival procedures. The cells obtained from a patient are cultured and used in an autogenous manner in the same patient for periodontal reconstruction. Yamada et al., for the first time, published their pioneering work on cell sheet engineering [4,5]. Recently, a more cost-effective approach to cell sheet formation, by culturing cells under the high-density condition on regular culture dishes, has been propounded and successively utilized [4,5,6]. The cell sheet technology has the potential to rectify the conundrum of scaffold fabrication [4,7,8]. The biggest setback of scaffold-based tissue engineering is the inflammatory response post-implantation, which hinders the utility of scaffolds and tissue engineering processes [9,10]. There is no need to employ synthetic biodegradable scaffolds in cell sheet technology, which thereby attenuates the risks of using scaffolds, and also reduces the post-transplantation immune response which can lead to graft rejection [11,12]. Cell sheet technology promotes the secretion of a matrix from cultured cells, which is feasible to harvest as an intact sheet [4,13,14]. By using this technique, the disbandment of isolated cells can be thwarted, even without using artificial scaffolds. This also helps in maintaining the desired shape by stacking the sheets of cell monolayers. Moreover, cell sheets show superior adhesion and ameliorated tissue regeneration. At the same time, cell sheets prevent unforeseen effects of the matrix as well as the action of proteolytic enzymes on surface molecules, hence representing a more innovative approach [4,7,11,15]. It has been understood that living cells, when used for the above-mentioned purpose, interact well with the host tissues modulating cytokine and growth factor expression and release, which heralds the process of wound healing and regeneration [16,17]. It is also understood that living cell constructs can be fabricated from stem cells under the influence and inducement of various chemical signals that can be administered in vitro. In this regard, quercetin is a molecule that has not been explored for its potential in inducing the formation of living cell constructs. Quercetin is a flavonoid molecule found in fruits, vegetables, grains, and wine [18]. It can be aptly termed a phytoestrogen, and has anti-inflammatory [19], antibacterial [20], and anticancer [21] properties.

Other than these properties, quercetin is also known to promote the proliferation and maintenance of stem cell integrity, without altering the stemness properties of the cells. The abovementioned effect of quercetin has been tested on human skin tissue stem cell cultures, where quercetin administration increased the proliferation rate of the stem cells through the estrogen receptor/β-catenin/c-Myc/cyclin A2 signaling pathway [22]. These functions compel us to explore the effects of quercetin on dental pulp stem cells. With the available background information, the present research study proposes the alternative use of living cell constructs, derived from dental pulp stem cells akin to scaffolds, consequently augmented with quercetin administration as a tool intended for soft tissue augmentation. It is reiterated that the principal objective behind the present study, although clinically not tested or implemented, is the fabrication of autogenous cell constructs, viz., obtaining dental pulp stem cells from a patient, followed by fabrication of living cell constructs under the influence of quercetin, for clinical use on the same patient.

## 2. Materials and Methods

The study protocol was approved by Scientific Research (IRB), College of Dentistry, Jazan University, Jazan, Saudi Arabia (CODJU-19702).

### 2.1. Sample Collection

Human third molar teeth were collected from healthy subjects, aged 14–25, undergoing orthodontic tooth extraction with appropriate oral hygiene (n = 5). Informed consent was obtained in accordance with institutional ethics considerations. The pulp tissue was extirpated from the extracted teeth using sterile and aseptic protocols, and directly transferred to the molecular biology laboratory for further processing.

### 2.2. Culture and Expansion of Human DPSCs

Isolation and characterization of DPSCs was carried out using the explant culture method described previously. Briefly, pulp tissue was minced into tiny fragments, and the pieces were placed in 35 mm polystyrene plastic culture dishes. A sufficient amount of fetal bovine serum (FBS) (Gibco, Rockville, MD, USA) was added to the tissues to cover them completely. A 24 h incubation at 37 °C and 5% CO_2_ was completed for explant tissue containing FBS; the whole DPSCs culture system was further maintained in DMEM (Invitrogen, Carlsbad, CA, USA), supplemented with 20% FBS and antibiotic-antimycotic solution at the same temperature and CO_2_ conditions. The culture medium was replenished twice weekly, and the cell growth, health, and morphology were monitored regularly with an inverted phase-contrast microscope. At 70–80% confluence, cells were detached using 0.25% Trypsin-EDTA solution (Invitrogen, Carlsbad, CA, USA) and transferred to a bigger, 25 cm^2^ polystyrene culture flask (Nunc, Rochester, NY, USA). Confluent DPSCs were detached using 0.25% Trypsin-EDTA solution and then continuously passaged in for expansion and further experiments. Cells from passage 2 to 4 were used in the experimentation.

### 2.3. Characterization of DPSCs Using Flow Cytometry

The confluent DPSCs were garnered for flow cytometry analysis. They were doused with phosphate buffer saline and incubated with anti-CD73-PE, anti-CD34-APC, anti-CD90-FITC, and anti-HLA-DR-PE (all monoclonal) antibodies (all from Invitrogen) for 30 min at 4 °C. Per sample, a minimum acquirement of 10,000 events was achieved. In comparison to the control, the calculation of the positive staining degree was inferred in percentage.

### 2.4. 3-(4,5-Dimethylthiazol-2-yl)-2,5-diphenyltetrazolium Bromide (MTT) Assay of DPSC Following Quercetin Treatment

MTT assay was employed to assess the cell viability for DPSCs treated with quercetin. The cells were seeded into 96-well plates (cell density per well was 5 × 10^3^ cells) and incubated with appropriate concentrations of quercetin (0.5 µM, 2 µM, 5 µM, 10 µM, 20 µM, and 50 µM) mixed with the complete medium (DMEM + 10% FBS) for 48 h. Following this, to individual wells, an addition of MTT (Sigma, St. Louis, MO, USA) solution of 0.5 mg/mL was made, and the plates were incubated for 4 h at 37 °C. Subsequently, following the removal of the medium, to the individual wells, 100 µL dimethyl sulfoxide (DMSO) (Sigma) was added. The measurement of absorbance was performed at 570 nm using a spectrophotometer.

### 2.5. Formation of Cell Constructs

The DPSCs were seeded in 35 mm tissue culture dishes and fed with the complete growth medium containing DMEM with 20% FBS, with or without 10 µM quercetin directly dissolved in DMEM for 15 days. The medium was changed every alternate day. The layer and cell constructs formed by the cells were then gently detached from the surface of the dishes with forceps.

### 2.6. Characterization of Living Cell Constructs

The cell constructs formed by the DPSCs were further subjected to experimental procedures to assess the histology of the cell sheets by hematoxylin and eosin staining.

### 2.7. Real-Time PCR for Analysis of Gene Expression

Using the Trizol RNA extraction method, the RNA was obtained from the pelleted cells fractionated from the cell sheets. cDNA synthesis kit was used for reverse transcribing and 1 μg RNA. Quantitative analysis of genes of interest presented in Table 1 along with the primer sequences was performed using SYBR Green PCR master mix on a quantitative RT-PCR system. Expression of target genes was normalized to a β-actin gene using the ΔΔCt method. The 2^−ΔΔCt^ method was employed to quantify the data on the relative gene expression. This was normalized to the average CT for the β-actin gene.

## 3. Results

**Figure 1:**
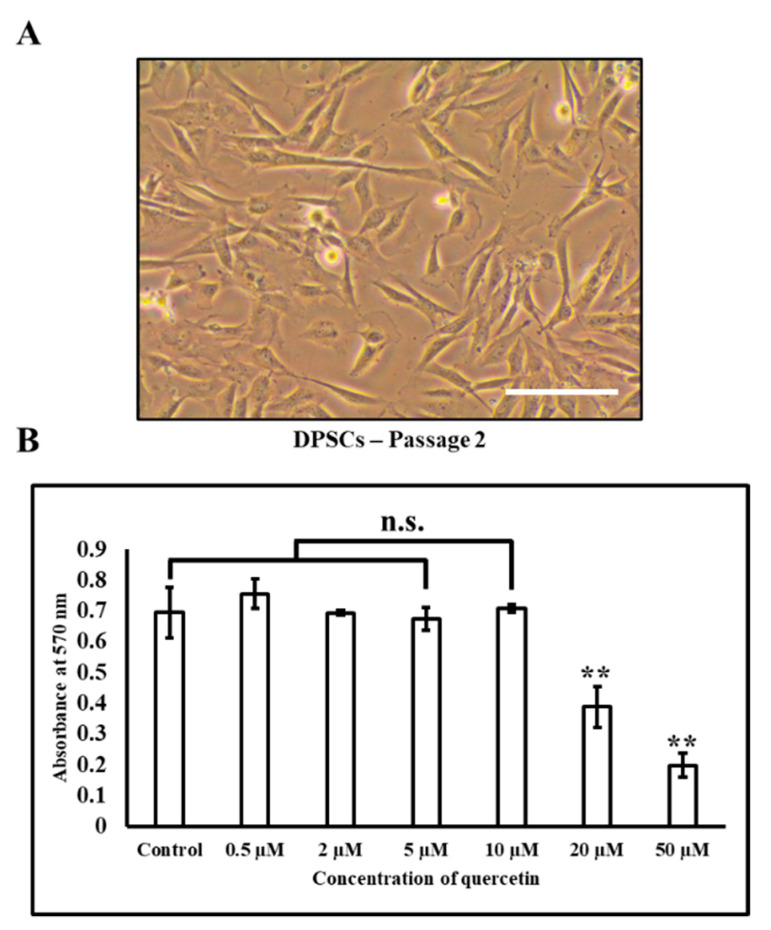
Characterization of DPSCs. (**A**) DPSCs at Passage 2. Scale bar = 100 µm; (**B**) The MTT assay. Concentrations including and below 10 µM of quercetin were found to be non-toxic to DPSCs. n.s. not significant. ** *p* < 0.001.

DPSCs were observed under a phase-contrast microscope for their morphological attributes. DPSC appeared elongated and spindle-shaped (Figure 1A). The growth of the cells was continuously monitored throughout the protocol duration. The cells looked healthy with some gradual morphological changes. There was no sign of cell death as no floating cells were observed, and the cells remained attached to the surface. The MTT assay was performed to check the viability of the DPSCs after treatment with quercetin. Concentrations including and below 10 µM of quercetin were found to be non-toxic to DPSCs (Figure 1B).

### 3.1. Assessment of Expression of Stem Cell Markers in the Dental Pulp Stem Cells by Flow Cytometry

**Figure 2:**
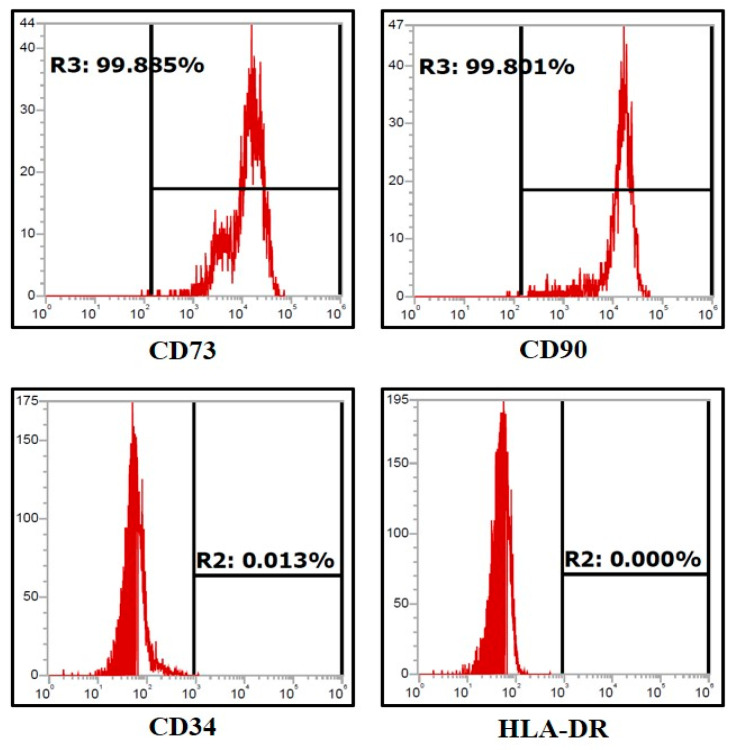
Flow cytometry analysis of DPSCs for MSC-specific cell surface markers (n = 5): CD73, CD90, CD34, and HLA-DR.

Expression of CD73 (>90%) and CD90 (>90%), the mesenchymal stem cell markers, were shown by the DPSCs (Figure 2); however, all the DPSCs did not show the expression of CD34, which is a hematopoietic stem cell marker, and HLA-DR, the MHC class-II cell surface receptor (Figure 2).

### 3.2. Effects of Quercetin Administration on Cell Sheet Characteristics and Physical Properties

**Figure 3:**
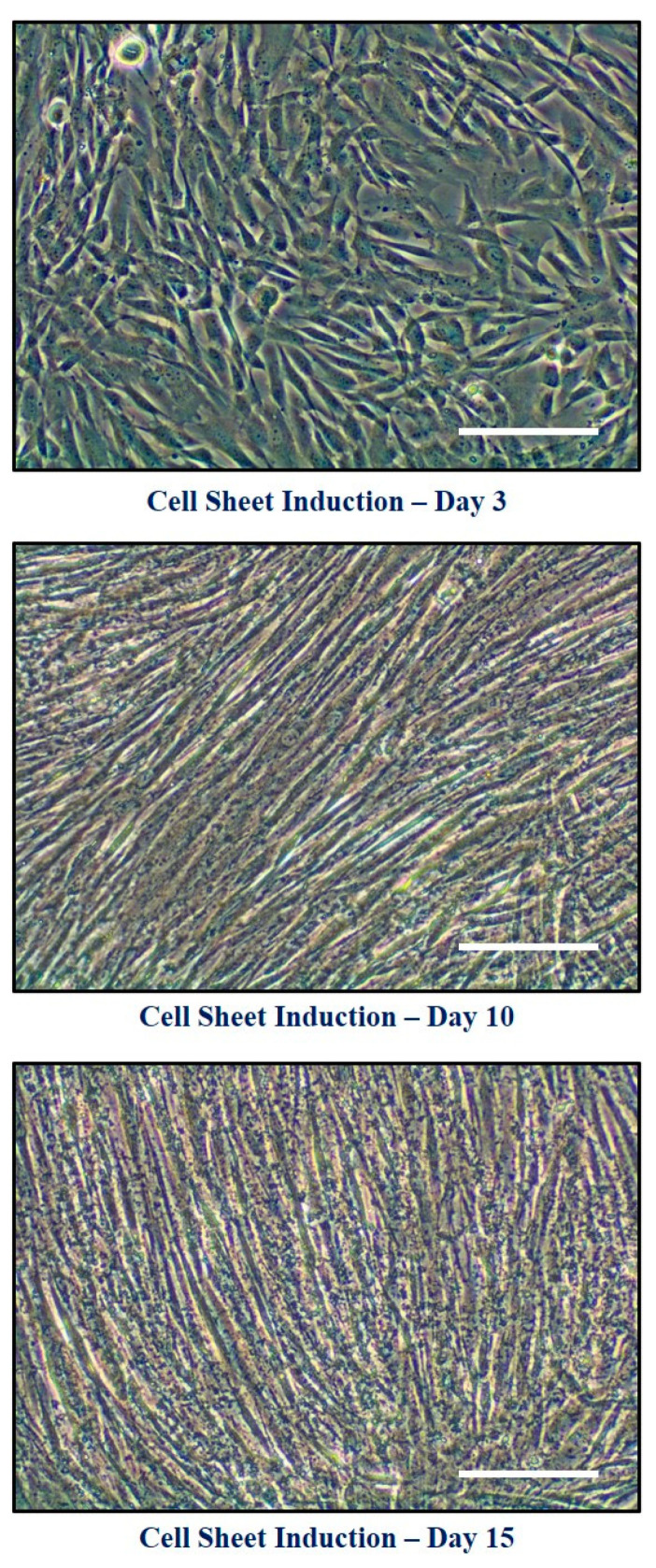
Morphology of DPSCs at different time points of cell sheet induction: Day 3, Day 10, and Day 15. Scale bar = 100 µm.

**Figure 4:**
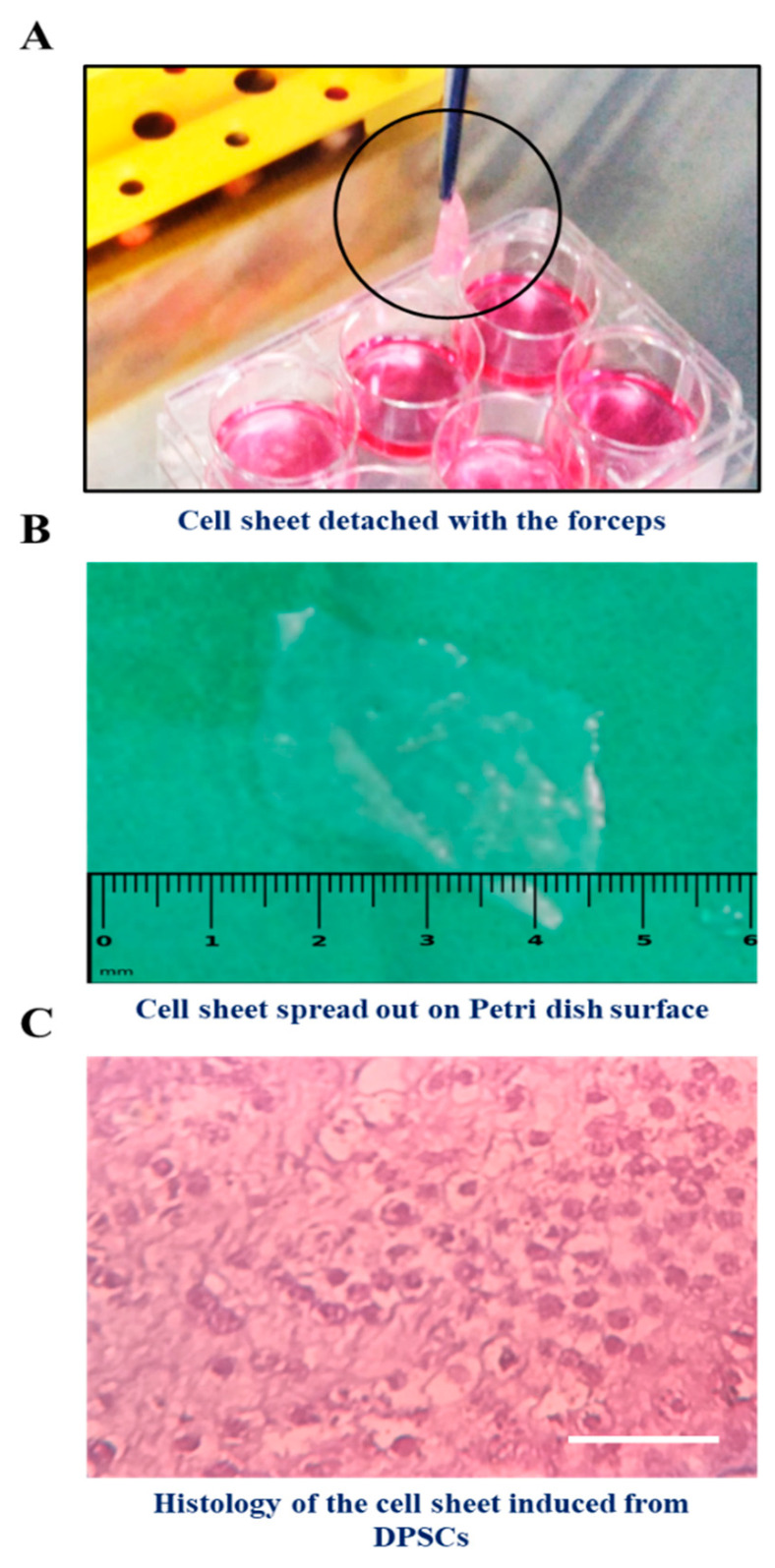
Acquisition and microscopic analysis of the cell constructs. (**A**) A cell construct detached from the surface of the plate; (**B**) DPSC–cell construct spread on a flat surface; (**C**) Histology of the cell construct with H & E staining.

The morphology of DPSCs at different time points of cell sheet induction (Day 3, Day 10, and Day 15) were assessed (Figure 3). The cell constructs induced from the DPSCs with the treatment of quercetin were easily detached from the surface of the plate with the forceps, whereas there was no formation in the control cells (without quercetin treatment) (Figure 4A). We observed that it was simple to transfer the cell construct on the flattened surface (Figure 4B). Histological characterization of the cell construct stained with hematoxylin and eosin showed that the sheet was intermingled with cells surrounding a tissue matrix (Figure 4C).

### 3.3. Assessment of Expression of Stemness-Related Genes in DPSCs with and without Quercetin Treatment

**Figure 5:**
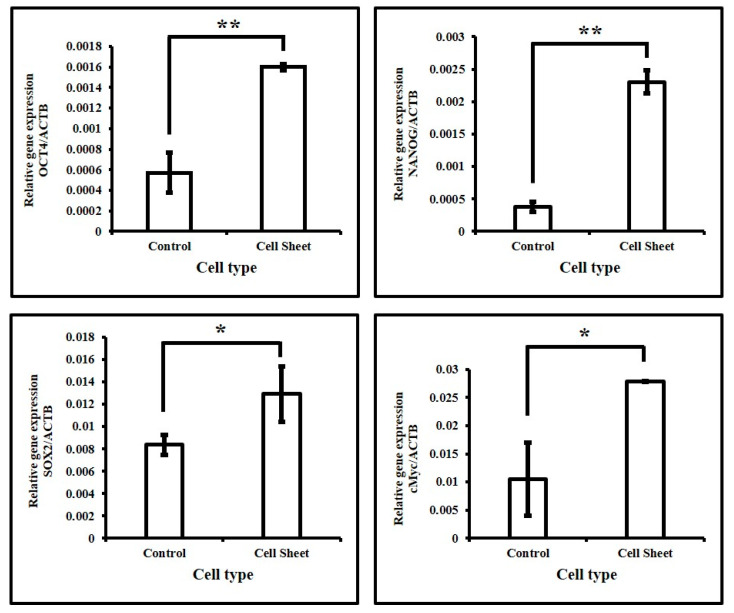
Gene expression analysis of stemness-related genes in DPSCs. Comparative gene expression analyses of OCT4, NANOG, SOX2, and cMyc in DPSCs before and after induction of cell sheet. * *p* < 0.05, ** *p* < 0.001.

The cells from the living construct were subjected to RNA isolation, and the same was conducted for DPSCs without treatment. The qRT-PCR analysis demonstrated that the expression of stemness-related genes OCT4, NANOG, SOX2, and cMyc was significantly increased with the use of quercetin (Figure 5). 

## 4. Discussion

The present study was performed to fabricate cell constructs, derived from human dental pulp stem cells, and cultured from explant tissues. The purpose was to form an exogenous living scaffold as an alternative to autogenous tissue grafts. A plethora of surgical techniques aimed at augmenting soft tissues around both natural teeth and dental implants have been documented in the literature. These procedures use the principles of periodontal plastic surgery and employ the use of soft tissue grafts obtained from the patient as an autograft from suitable donor sites [23,24]. The preferred donor site for graft harvest is mainly the hard palate from which both free gingival autografts and free connective tissue autografts can be obtained. However, these techniques, even though they are associated with moderate predictability, are associated with other combined adverse effects such as severe pain and ulceration in the donor site, and aesthetic concerns such as color match discrepancy with the neighboring grafted sites, and so on. To circumvent these difficulties, tissue engineering and regenerative medicine techniques have employed the use of scaffolds that have biomimetic properties and can be used in soft tissue augmentation [25,26]. There are also commercially available scaffolds obtained from various sources such as AlloDerm, which are classically described as human-derived acellular dermal matrices. The problem which can be anticipated with synthetic scaffolds, termed alloplasts, is a lack of biocompatibility. Moreover, the lack of presence of living cells in a viable state in these alloplastic scaffolds reduces their regenerative potential. Concerning xenograft materials, there is an associated risk of prion-induced diseases which can occur with long-term use [27]. It is also noted that cultural and social concerns may reduce the acceptability of animal-derived products for many patients undergoing periodontal regenerative procedures.

Today can be rightly called the era of stem cell advances. Stem cells can be obtained from various sources such as blood, umbilical cord, bone marrow, and human teeth, and are broadly hematopoietic or mesenchymal in origin. Mesenchymal stem cells (MSCs) have been shown to improve wound healing and regenerate tissue owing to their paracrine secretions and differentiation ability [28,29]. Various organs and connective tissues comprising fat tissue, umbilical cord, bone marrow, dental pulp, and skin can be used to retrieve or isolate MSCs [4,28,29,30,31]. Dental stem cells, a particular type of MSC, have been retrieved from the periodontal ligament, apical papilla, gingiva, dental follicle, and dental pulp [29,30]. Each of these sources can give rise to another, different type of dental stem cell. Stem cells from dental pulp (DPSCs) are exclusive since these are of neural crest origin and possess the aptitude for self-replenishment and differentiation into many specialized cell lineages [29]. Human DPSCs express a specific set of surface markers that are exclusively expressed by embryonic stem cells (ESCs) and MSCs [7,29,31]. Furthermore, DPSCs have been revealed to demonstrate a great aptitude for bone or dentin construction in colony-derived clones after being introduced to animal models [29,30,31,32]. The DPSCs could provide a valued reserve for cell replacement therapeutics and tissue engineering. Besides this, there are very narrow legal and ethical concerns about the therapeutic practice of the DPSCs, hence the accumulated attention on DPSCs for regenerative biology research [33]. Human dental pulp tissue is rich in stem cells and can be easily harvested from extracted healthy human teeth. Cell sheet technology is another advance in the field of stem cell biology. A recent study [7] has used this technology to fabricate dental pulp stem cell sheets for bone regeneration. It is noteworthy that this study used a clonogenic medium containing vitamin C. The present study had a similar aim of fabricating dental pulp stem cell, living constructs. As a part of this pursuit, pulp tissue was obtained from human teeth, extracted for orthodontic purposes. The pulp tissue was subjected to maceration, and the stem cells isolated from the tissues were cultured and expanded in vitro. To characterize the same as stem cells, the flow cytometry technique was used with an array of markers. The results revealed that the cells expressed CD73 (>90%) and CD90 (>90%), the mesenchymal stem cell markers, and did not express CD34, the hematopoietic stem cell marker. This finding is of paramount importance to ascertain that the cells were mesenchymal stem cells and were not hematopoietic in origin. Furthermore, these cells were cultured in a medium containing quercetin. It was found by MTT assay that quercetin concentrations <10 µM were non-toxic to the DPSCs. Concerning stem cells, quercetin has been shown to increase the proliferation rate of epidermal stem cells in an in vitro skin culture model [22]. These results demonstrate the positive effects of quercetin in stem cell biology. However, this molecule has not been used in the fabrication of living stem cell constructs. To explore this, the present study administered quercetin to the stem cells during the cell construct fabrication process. This addition yielded promising results. The mechanical properties of the cell constructs were better in the present study, as manipulation of the sheet was easier in the quercetin group, compared to the control cells that were cultured without quercetin administration. A hematoxylin and eosin staining process revealed the presence of an extracellular matrix with significant content in the cell sheets. Furthermore, a genetic analysis by PCR technique revealed an elevation in stemness-related genes under the influence of quercetin. Previous studies have reported that there is an expression of chondrogenesis-related genes in the stem cells after cell sheet formation [10,11]. The stem cells start differentiating after the cell sheet induction. However, with the use of quercetin, this hurdle could be overcome. It is always considered a necessary task for a stem cell to maintain its pluripotency while multiplying in number [7,30], in order for the stem cells to further grow and fulfill their functions in the transplanted environment. The findings of the present study shed light on the use of a modality, termed bioengineered live cellular therapy, which precisely denotes the use of live cell constructs to achieve the regeneration of soft tissues. Previous studies have used constructs with living keratinocytes and fibroblasts, obtained from oral tissues, for soft tissue augmentation [34,35]. The primary advantage of using living cell constructs as modalities for attempting tissue repair is mainly based on the fact that these constructs, even though composed of live cells, do not act like autografts. When they are placed in the surgical site, they aid orchestrated healing by timed production and release of growth factors and cytokines, such as vascular endothelial growth factor. This stimulates angiogenesis and fosters the cells in the wound microenvironment to differentiate into an array of different cell types in a timed manner for complete regeneration [16]. The present study is novel; a live cell construct composed of DPSC has been successfully initiated and carried out under the influence of quercetin. The multifaceted properties of quercetin make this molecule ideal for use in aiding the formation of dental pulp stem cell living constructs. These constructs are predominantly intended for use in periodontal plastic surgical procedures, where they would be grafted onto the gingival recession sites to regenerate the lost gingiva. The significant hurdle associated with the procedure is the need for tooth extraction to isolate and expand dental pulp stem cells. This may be a concern and may have reduced acceptance among patients who may defer dental extraction. However, considering the benefits over the risks, there could be more patients in the future opting for such novel treatment modalities.

## 5. Conclusions

In the present study, quercetin was shown to successfully initiate living cell constructs in DPSCs, under specific nutritional supplementation. The procedure of obtaining DPSCs and subsequent fabrication of the living cell constructs takes approximately 2 weeks. In terms of timing, the procedure is highly clinically feasible. However, these protocols need to be further optimized to achieve tailor-made living cell constructs within a shorter period, for applications in regenerative medicine. This technique is easy to apply and is a promising alternative to current treatments. The present research is in a nascent stage and has not yet been applied on patients. The future target would be to apply the living cell constructs successfully on patients needing periodontal plastic surgery. If fruitful results are obtained, it will be easy and feasible to use these cell constructs for autogenous soft tissue augmentation in dentistry.

## Figures and Tables

**Table 1:** List of primers.

Gene	Forward Primer	Reverse Primer
NANOG	5′-TTT GTG GGC CTG AAG AAA ACT-3′	5′-AGG GCT GTC CTG AAT AAG CAG-3′
OCT4	5′-GTG GAG GAA GCT GAC AAC AA-3′	5′-ATT CTC CAG GTT GCC TCT CA-3′
cMyc	5′-AGA AAT GTC CTG AGC AAT CAC C-3′	5′-AAG GTT GTG AGG TTG CAT TTG A-3′
SOX2	5′-CCA GCA GAC TTC ACA TGT CC-3′	5′-ACA TGT GTG AGA GGG GCA GT-3′
ACTIN	5′-AGA GCT ACG AGC TGC CTG AC-3′	5′-AGC ACT GTG TTG GCG TAC AG-3′

## References

[1] Helderman W.H.V.P., Lembariti B., Van Der Weijden G.A., Hof M.A.V. (1998). T Gingival recession and its association with calculus in subjects deprived of prophylactic dental care. J. Clin. Periodontol..

[2] Khocht A., Simon G., Person P., Denepitiya J.L. (1993). Gingival Recession in Relation to History of Hard Toothbrush Use. J. Periodontol..

[3] Gunsolley J., Quinn S., Tew J., Gooss C., Brooks C., Schenkein H. (1998). The Effect of Smoking on Individuals with Minimal Periodontal Destruction. J. Periodontol..

[4] Honjo K.-I., Yamamoto T., Adachi T., Amemiya T., Mazda O., Kanamura N., Kita M. (2015). Evaluation of a dental pulp-derived cell sheet cultured on amniotic membrane substrate. Bio-Med. Mater. Eng..

[5] Yuan Z., Min J., Zhao Y., Cheng Q., Wang K., Lin S., Luo J., Liu H. (2018). Quercetin rescued TNF-alpha-induced impairments in bone marrow-derived mesenchymal stem cell osteogenesis and improved osteoporosis in rats. Am. J. Transl. Res..

[6] Li Y., Wang J., Chen G., Feng S., Wang P., Zhu X., Zhang R. (2015). Quercetin promotes the osteogenic differentiation of rat mesenchymal stem cells via mitogen-activated protein kinase signaling. Exp. Ther. Med..

[7] Pedroni A.C.F., Sarra G., De Oliveira N.K., Moreira M.S., Deboni M.C., Marques M.M. (2018). Cell sheets of human dental pulp stem cells for future application in bone replacement. Clin. Oral Investig..

[8] Patil V.R., Kharat A.H., Kulkarni D.G., Kheur S.M., Bhonde R.R. (2018). Long term explant culture for harvesting homogeneous population of human dental pulp stem cells. Cell Biol. Int..

[9] Miladpour B., Rasti M., Owji A.A., Mostafavipour Z., Khoshdel Z., Noorafshan A., Zal F. (2016). Quercetin potentiates transdifferentiation of bone marrow mesenchymal stem cells into the beta cells in vitro. J. Endocrinol. Investig..

[10] Pang X.-G., Cong Y., Bao N.-R., Li Y.-G., Zhao J.-N. (2018). Quercetin Stimulates Bone Marrow Mesenchymal Stem Cell Differentiation through an Estrogen Receptor-Mediated Pathway. BioMed Res. Int..

[11] Baral S., Pariyar R., Kim J., Lee H.-S., Seo J. (2017). Quercetin-3-O-glucuronide promotes the proliferation and migration of neural stem cells. Neurobiol. Aging.

[12] Hu L., Zhao B., Gao Z., Xu J., Fan Z., Zhang C., Wang J., Wang S. (2020). Regeneration characteristics of different dental derived stem cell sheets. J. Oral Rehabil..

[13] Pedroni A.C., Diniz I.M., Abe G.L., Moreira M.S., Sipert C.R., Marques M.M. (2018). Photobiomodulation therapy and vitamin C on longevity of cell sheets of human dental pulp stem cells. J. Cell. Physiol..

[14] Itoh Y., Sasaki J., Hashimoto M., Katata C., Hayashi M., Imazato S. (2018). Pulp Regeneration by 3-dimensional Dental Pulp Stem Cell Constructs. J. Dent. Res..

[15] Na S., Zhang H., Huang F., Wang W., Ding Y., Li D., Jin Y. (2016). Regeneration of dental pulp/dentine complex with a three-dimensional and scaffold-free stem-cell sheet-derived pellet. J. Tissue Eng. Regen. Med..

[16] McGuire M.K., Scheyer E.T., Nevins M.L., Neiva R., Cochran D.L., Mellonig J.T., Giannobile W.V., Bates D. (2011). Living Cellular Construct for Increasing the Width of Keratinized Gingiva: Results from a Randomized, Within-Patient, Controlled Trial. J. Periodontol..

[17] McGuire M.K., Scheyer E.T., Nunn M.E., Lavin P.T. (2008). A Pilot Study to Evaluate a Tissue-Engineered Bilayered Cell Therapy as an Alternative to Tissue from the Palate. J. Periodontol..

[18] Parasuraman S., David A.V.A., Arulmoli R. (2016). Overviews of biological importance of quercetin: A bioactive flavonoid. Pharmacogn. Rev..

[19] Xiao X., Shi D., Liu L., Wang J., Xie X., Kang T., Deng W. (2011). Quercetin Suppresses Cyclooxygenase-2 Expression and Angiogenesis through Inactivation of P300 Signaling. PLoS ONE.

[20] Rigano D., Formisano C., Basile A., Lavitola A., Senatore F., Rosselli S., Bruno M. (2007). Antibacterial activity of flavonoids and phenylpropanoids from Marrubium globosumssp. libanoticum. Phytother. Res..

[21] Hashemzaei M., Far A.D., Yari A., Heravi R.E., Tabrizian K., Taghdisi S.M., Sadegh S.E., Tsarouhas K., Kouretas D., Tzanakakis G. (2017). Anticancer and apoptosis-inducing effects of quercetin in vitro and in vivo. Oncol. Rep..

[22] Wang Z., Zhang G., Le Y., Ju J., Zhang P., Wan D., Zhao Q., Jin G., Su H., Liu J. (2020). Quercetin promotes human epidermal stem cell proliferation through the estrogen receptor/β-catenin/c-Myc/cyclin A2 signaling pathway. Acta Biochim. Biophys. Sin..

[23] Zuhr O., Bäumer D., Hürzeler M. (2014). The addition of soft tissue replacement grafts in plastic periodontal and implant surgery: Critical elements in design and execution. J. Clin. Periodontol..

[24] Zucchelli G., Tavelli L., McGuire M.K., Rasperini G., Feinberg S.E., Wang H., Giannobile W.V. (2019). Autogenous soft tissue grafting for periodontal and peri-implant plastic surgical reconstruction. J. Periodontol..

[25] Larsson L., Decker A., Nibali L., Pilipchuk S., Berglundh T., Giannobile W. (2016). Regenerative Medicine for Periodontal and Peri-implant Diseases. J. Dent. Res..

[26] Han J., Menicanin D., Gronthos S., Bartold P.M. (2013). Stem cells, tissue engineering and periodontal regeneration. Aust. Dent. J..

[27] Nunery W.R. (2001). Risk of Prion Transmission with the Use of Xenografts and Allografts in Surgery. Ophthalmic Plast. Reconstr. Surg..

[28] Monteiro B.G., Serafim R.C., Melo G.B., Silva M.C.P., Lizier N.F., Maranduba C.M.C., Smith R.L., Kerkis A., Cerruti H., Gomes J.A.P. (2009). Human immature dental pulp stem cells share key characteristic features with limbal stem cells. Cell Prolif..

[29] Meng M.H., Hu L., Zhou Y., Ge Z., Wang H., Wu C.-T., Jin J. (2020). A Sandwich Structure of Human Dental Pulp Stem Cell Sheet, Treated Dentin Matrix, and Matrigel for Tooth Root Regeneration. Stem Cells Dev..

[30] Fujii Y., Kawase-Koga Y., Hojo H., Yano F., Sato M., Chung U.-I., Ohba S., Chikazu D. (2018). Bone regeneration by human dental pulp stem cells using a helioxanthin derivative and cell-sheet technology. Stem Cell Res. Ther..

[31] Monteiro N., Smith E.E., Angstadt S., Zhang W., Khademhosseini A., Yelick P.C. (2016). Dental cell sheet biomimetic tooth bud model. Biomaterials.

[32] Garrido P., Pedroni A., Cury D., Moreira M., Rosin F., Sarra G., Marques M. (2019). Effects of photobiomodulation therapy on the extracellular matrix of human dental pulp cell sheets. J. Photochem. Photobiol. B Biol..

[33] Casado-Díaz A., Anter J., Dorado G., Quesada-Gómez J.M. (2016). Effects of quercetin, a natural phenolic compound, in the differentiation of human mesenchymal stem cells (MSC) into adipocytes and osteoblasts. J. Nutr. Biochem..

[34] Izumi K., Terashi H., Marcelo C., Feinberg S. (2000). Development and characterization of a tissue-engineered human oral mucosa equivalent produced in a serum-free culture system. J. Dent. Res..

[35] Izumi K., Takacs G., Terashi H., Feinberg S.E. (1999). Ex vivo development of a composite human oral mucosal equivalent. J. Oral Maxillofac. Surg..

